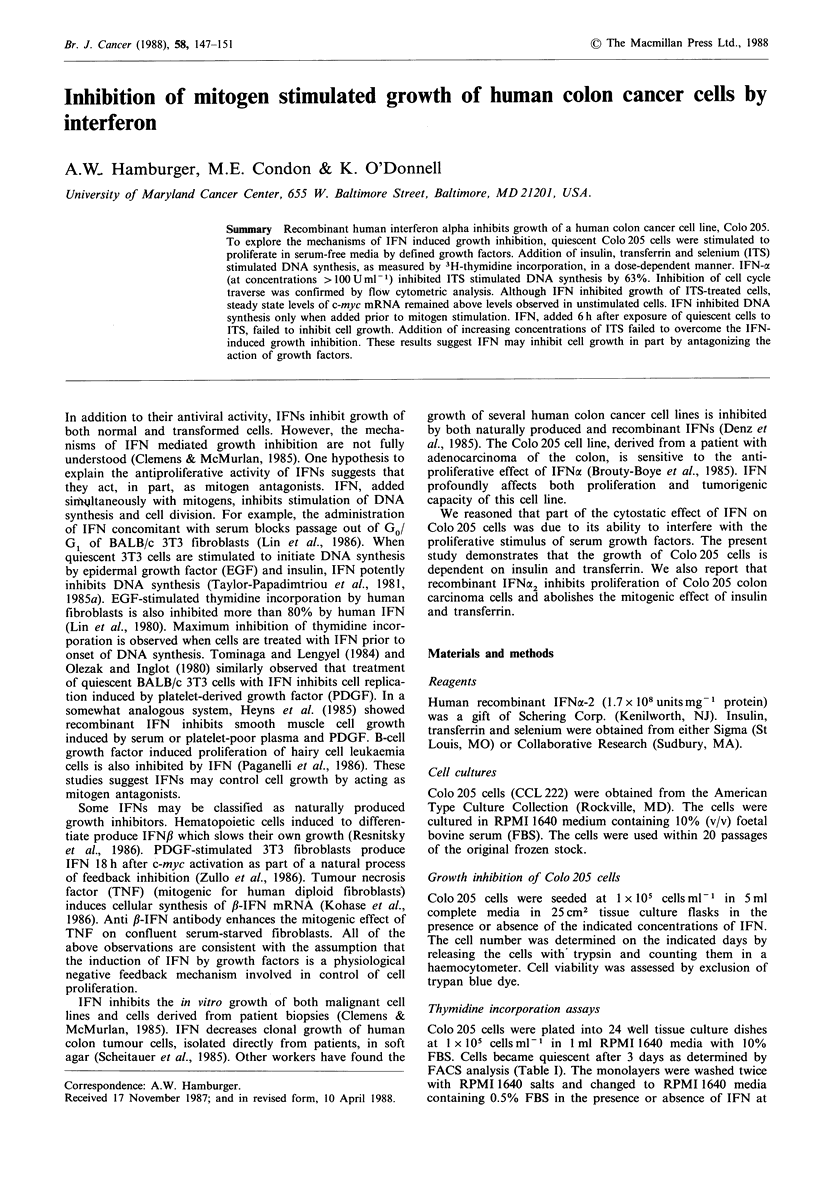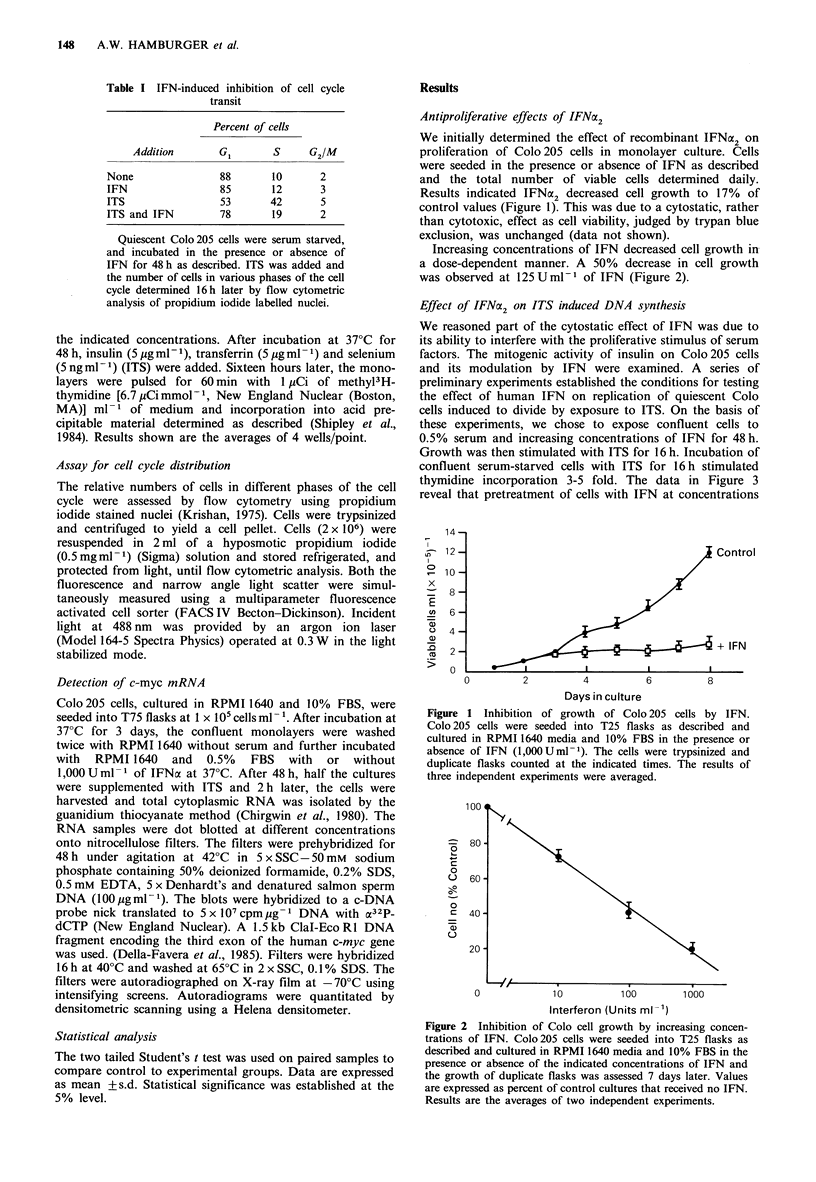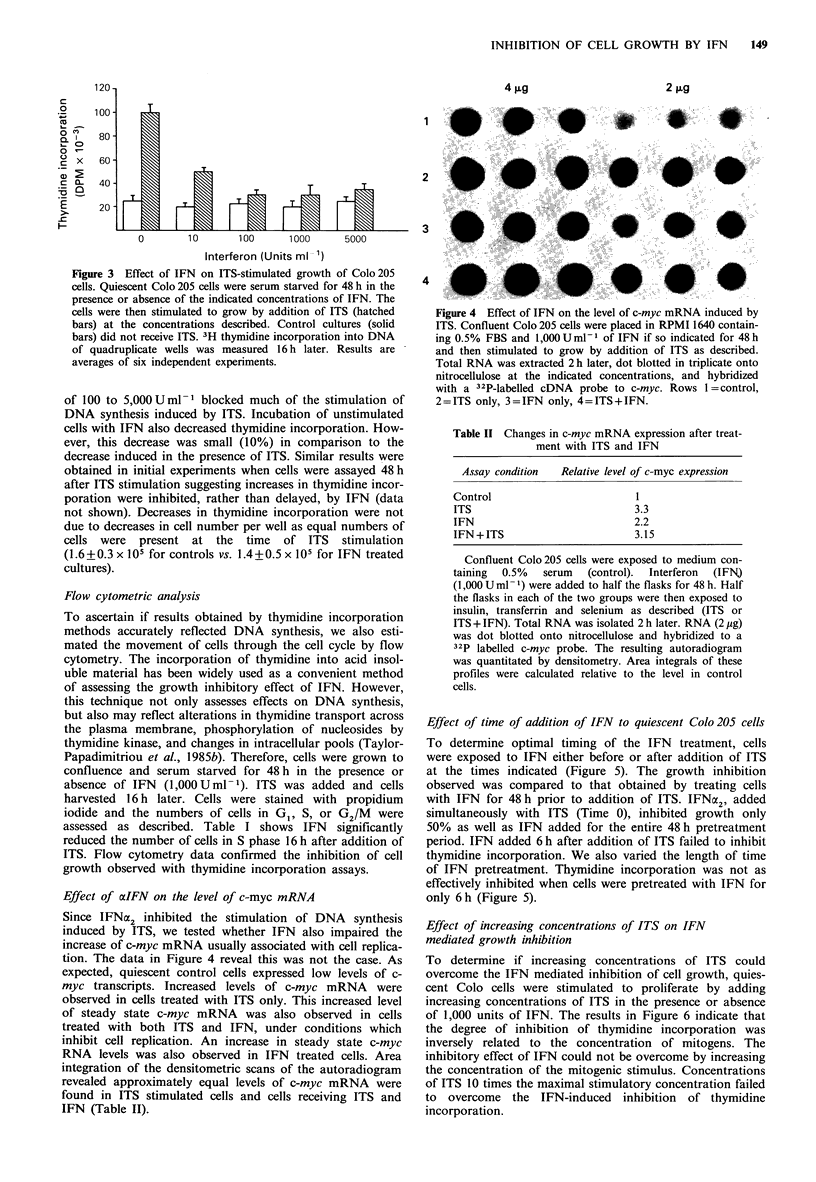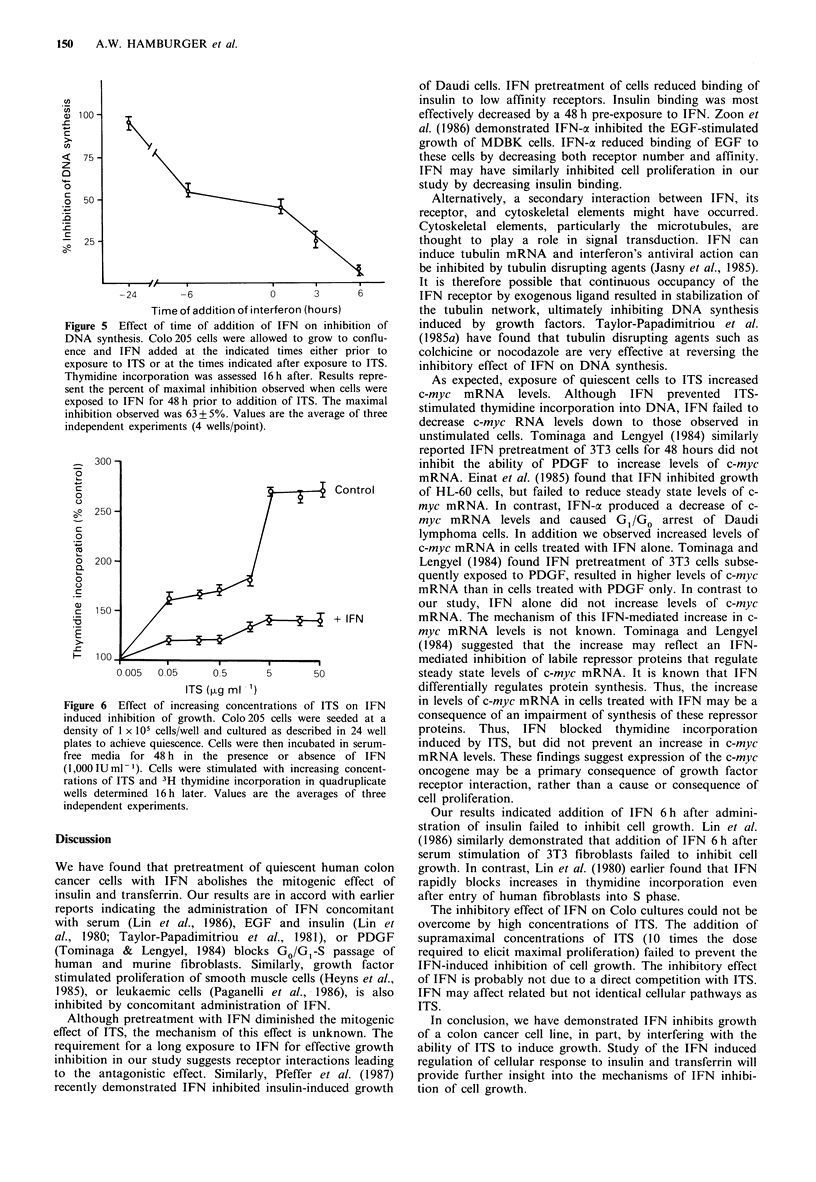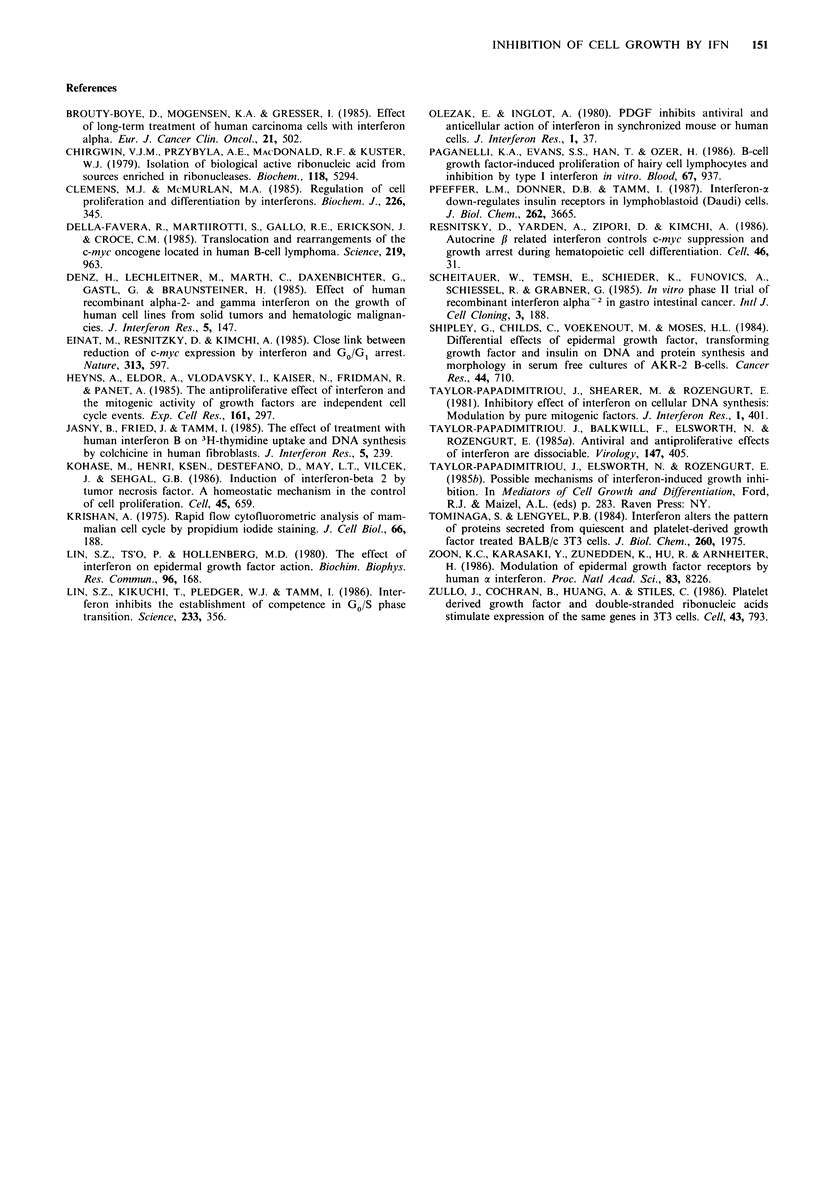# Inhibition of mitogen stimulated growth of human colon cancer cells by interferon.

**DOI:** 10.1038/bjc.1988.182

**Published:** 1988-08

**Authors:** A. W. Hamburger, M. E. Condon, K. O'Donnell

**Affiliations:** University of Maryland Cancer Center, Baltimore 21201.

## Abstract

**Images:**


*Br. J. Cancer* (1988), **58**, 147–151 © The Macmillan Press Ltd., 1988

### Inhibition of mitogen stimulated growth of human colon cancer cells by interferon

A.W. Hamburger, M.E. Condon & K. O'Donnell

*University of Maryland Cancer Center, 655 W. Baltimore Street, Baltimore, MD 21201, USA.*

**Summary** Recombinant human interferon alpha inhibits growth of a human colon cancer cell line, Colo 205. To explore the mechanisms of IFN induced growth inhibition, quiescent Colo 205 cells were stimulated to proliferate in serum-free media by defined growth factors. Addition of insulin, transferrin and selenium (ITS) stimulated DNA synthesis, as measured by $^3$H-thymidine incorporation, in a dose-dependent manner. IFN-α (at concentrations $>100\,\mathrm{U\,ml^{-1}}$) inhibited ITS stimulated DNA synthesis by 63%. Inhibition of cell cycle traverse was confirmed by flow cytometric analysis. Although IFN inhibited growth of ITS-treated cells, steady state levels of c-*myc* mRNA remained above levels observed in unstimulated cells. IFN inhibited DNA synthesis only when added prior to mitogen stimulation. IFN, added 6 h after exposure of quiescent cells to ITS, failed to inhibit cell growth. Addition of increasing concentrations of ITS failed to overcome the IFN-induced growth inhibition. These results suggest IFN may inhibit cell growth in part by antagonizing the action of growth factors.

In addition to their antiviral activity, IFNs inhibit growth of both normal and transformed cells. However, the mechanisms of IFN mediated growth inhibition are not fully understood (Clemens & McMurlan, 1985). One hypothesis to explain the antiproliferative activity of IFNs suggests that they act, in part, as mitogen antagonists. IFN, added simultaneously with mitogens, inhibits stimulation of DNA synthesis and cell division. For example, the administration of IFN concomitant with serum blocks passage out of $G_0$/$G_1$ of BALB/c 3T3 fibroblasts (Lin *et al.*, 1986). When quiescent 3T3 cells are stimulated to initiate DNA synthesis by epidermal growth factor (EGF) and insulin, IFN potently inhibits DNA synthesis (Taylor-Papadimtriou *et al.*, 1981, 1985*a*). EGF-stimulated thymidine incorporation by human fibroblasts is also inhibited more than 80% by human IFN (Lin *et al.*, 1980). Maximum inhibition of thymidine incorporation is observed when cells are treated with IFN prior to onset of DNA synthesis. Tominaga and Lengyel (1984) and Olezak and Inglot (1980) similarly observed that treatment of quiescent BALB/c 3T3 cells with IFN inhibits cell replication induced by platelet-derived growth factor (PDGF). In a somewhat analogous system, Heyns *et al.* (1985) showed recombinant IFN inhibits smooth muscle cell growth induced by serum or platelet-poor plasma and PDGF. B-cell growth factor induced proliferation of hairy cell leukaemia cells is also inhibited by IFN (Paganelli *et al.*, 1986). These studies suggest IFNs may control cell growth by acting as mitogen antagonists.

Some IFNs may be classified as naturally produced growth inhibitors. Hematopoietic cells induced to differentiate produce IFNβ which slows their own growth (Resnitsky *et al.*, 1986). PDGF-stimulated 3T3 fibroblasts produce IFN 18 h after c-*myc* activation as part of a natural process of feedback inhibition (Zullo *et al.*, 1986). Tumour necrosis factor (TNF) (mitogenic for human diploid fibroblasts) induces cellular synthesis of β-IFN mRNA (Kohase *et al.*, 1986). Anti β-IFN antibody enhances the mitogenic effect of TNF on confluent serum-starved fibroblasts. All of the above observations are consistent with the assumption that the induction of IFN by growth factors is a physiological negative feedback mechanism involved in control of cell proliferation.

IFN inhibits the *in vitro* growth of both malignant cell lines and cells derived from patient biopsies (Clemens & McMurlan, 1985). IFN decreases clonal growth of human colon tumour cells, isolated directly from patients, in soft agar (Scheitauer *et al.*, 1985). Other workers have found the growth of several human colon cancer cell lines is inhibited by both naturally produced and recombinant IFNs (Denz *et al.*, 1985). The Colo 205 cell line, derived from a patient with adenocarcinoma of the colon, is sensitive to the antiproliferative effect of IFNα (Brouty-Boye *et al.*, 1985). IFN profoundly affects both proliferation and tumorigenic capacity of this cell line.

We reasoned that part of the cytostatic effect of IFN on Colo 205 cells was due to its ability to interfere with the proliferative stimulus of serum growth factors. The present study demonstrates that the growth of Colo 205 cells is dependent on insulin and transferrin. We also report that recombinant IFN$\alpha_2$ inhibits proliferation of Colo 205 colon carcinoma cells and abolishes the mitogenic effect of insulin and transferrin.

### Materials and methods

### *Reagents*

Human recombinant IFNα-2 ($1.7\times10^8$ units mg$^{-1}$ protein) was a gift of Schering Corp. (Kenilworth, NJ). Insulin, transferrin and selenium were obtained from either Sigma (St Louis, MO) or Collaborative Research (Sudbury, MA).

### *Cell cultures*

Colo 205 cells (CCL 222) were obtained from the American Type Culture Collection (Rockville, MD). The cells were cultured in RPMI 1640 medium containing 10% (v/v) foetal bovine serum (FBS). The cells were used within 20 passages of the original frozen stock.

### *Growth inhibition of Colo 205 cells*

Colo 205 cells were seeded at $1\times10^5$ cells ml$^{-1}$ in 5 ml complete media in 25 cm$^2$ tissue culture flasks in the presence or absence of the indicated concentrations of IFN. The cell number was determined on the indicated days by releasing the cells with trypsin and counting them in a haemocytometer. Cell viability was assessed by exclusion of trypan blue dye.

### *Thymidine incorporation assays*

Colo 205 cells were plated into 24 well tissue culture dishes at $1\times10^5$ cells ml$^{-1}$ in 1 ml RPMI 1640 media with 10% FBS. Cells became quiescent after 3 days as determined by FACS analysis (Table I). The monolayers were washed twice with RPMI 1640 salts and changed to RPMI 1640 media containing 0.5% FBS in the presence or absence of IFN at

Correspondence: A.W. Hamburger.
Received 17 November 1987; and in revised form, 10 April 1988.

**Table I** IFN-induced inhibition of cell cycle transit

| | *Percent of cells* | | |
|---|---|---|---|
| *Addition* | $G_1$ | *S* | $G_2/M$ |
| None | 88 | 10 | 2 |
| IFN | 85 | 12 | 3 |
| ITS | 53 | 42 | 5 |
| ITS and IFN | 78 | 19 | 2 |

Quiescent Colo 205 cells were serum starved, and incubated in the presence or absence of IFN for 48 h as described. ITS was added and the number of cells in various phases of the cell cycle determined 16 h later by flow cytometric analysis of propidium iodide labelled nuclei.

the indicated concentrations. After incubation at 37°C for 48 h, insulin (5 $\mu g\,ml^{-1}$), transferrin (5 $\mu g\,ml^{-1}$) and selenium (5 $ng\,ml^{-1}$) (ITS) were added. Sixteen hours later, the monolayers were pulsed for 60 min with 1 $\mu$Ci of methyl$^3$H-thymidine [6.7 $\mu Ci\,mmol^{-1}$, New England Nuclear (Boston, MA)] $ml^{-1}$ of medium and incorporation into acid precipitable material determined as described (Shipley *et al.*, 1984). Results shown are the averages of 4 wells/point.

### *Assay for cell cycle distribution*

The relative numbers of cells in different phases of the cell cycle were assessed by flow cytometry using propidium iodide stained nuclei (Krishan, 1975). Cells were trypsinized and centrifuged to yield a cell pellet. Cells ($2 \times 10^6$) were resuspended in 2 ml of a hyposmotic propidium iodide (0.5 $mg\,ml^{-1}$) (Sigma) solution and stored refrigerated, and protected from light, until flow cytometric analysis. Both the fluorescence and narrow angle light scatter were simultaneously measured using a multiparameter fluorescence activated cell sorter (FACS IV Becton–Dickinson). Incident light at 488 nm was provided by an argon ion laser (Model 164-5 Spectra Physics) operated at 0.3 W in the light stabilized mode.

### *Detection of c-*myc *mRNA*

Colo 205 cells, cultured in RPMI 1640 and 10% FBS, were seeded into T75 flasks at $1 \times 10^5$ cells $ml^{-1}$. After incubation at 37°C for 3 days, the confluent monolayers were washed twice with RPMI 1640 without serum and further incubated with RPMI 1640 and 0.5% FBS with or without 1,000 U $ml^{-1}$ of IFN$\alpha$ at 37°C. After 48 h, half the cultures were supplemented with ITS and 2 h later, the cells were harvested and total cytoplasmic RNA was isolated by the guanidium thiocyanate method (Chirgwin *et al.*, 1980). The RNA samples were dot blotted at different concentrations onto nitrocellulose filters. The filters were prehybridized for 48 h under agitation at 42°C in 5 x SSC – 50 mM sodium phosphate containing 50% deionized formamide, 0.2% SDS, 0.5 mM EDTA, 5 x Denhardt's and denatured salmon sperm DNA (100 $\mu g\,ml^{-1}$). The blots were hybridized to a c-DNA probe nick translated to $5 \times 10^7$ cpm $\mu g^{-1}$ DNA with $\alpha^{32}$P-dCTP (New England Nuclear). A 1.5 kb ClaI-Eco R1 DNA fragment encoding the third exon of the human c-*myc* gene was used. (Della-Favera *et al.*, 1985). Filters were hybridized 16 h at 40°C and washed at 65°C in 2 x SSC, 0.1% SDS. The filters were autoradiographed on X-ray film at –70°C using intensifying screens. Autoradiograms were quantitated by densitometric scanning using a Helena densitometer.

### *Statistical analysis*

The two tailed Student's *t* test was used on paired samples to compare control to experimental groups. Data are expressed as mean ±s.d. Statistical significance was established at the 5% level.

### Results

### *Antiproliferative effects of IFN*$\alpha_2$

We initially determined the effect of recombinant IFN$\alpha_2$ on proliferation of Colo 205 cells in monolayer culture. Cells were seeded in the presence or absence of IFN as described and the total number of viable cells determined daily. Results indicated IFN$\alpha_2$ decreased cell growth to 17% of control values (Figure 1). This was due to a cytostatic, rather than cytotoxic, effect as cell viability, judged by trypan blue exclusion, was unchanged (data not shown).

Increasing concentrations of IFN decreased cell growth in a dose-dependent manner. A 50% decrease in cell growth was observed at 125 U $ml^{-1}$ of IFN (Figure 2).

### *Effect of IFN*$\alpha_2$ *on ITS induced DNA synthesis*

We reasoned part of the cytostatic effect of IFN was due to its ability to interfere with the proliferative stimulus of serum factors. The mitogenic activity of insulin on Colo 205 cells and its modulation by IFN were examined. A series of preliminary experiments established the conditions for testing the effect of human IFN on replication of quiescent Colo cells induced to divide by exposure to ITS. On the basis of these experiments, we chose to expose confluent cells to 0.5% serum and increasing concentrations of IFN for 48 h. Growth was then stimulated with ITS for 16 h. Incubation of confluent serum-starved cells with ITS for 16 h stimulated thymidine incorporation 3-5 fold. The data in Figure 3 reveal that pretreatment of cells with IFN at concentrations

**Figure 1** Inhibition of growth of Colo 205 cells by IFN. Colo 205 cells were seeded into T25 flasks as described and cultured in RPMI 1640 media and 10% FBS in the presence or absence of IFN (1,000 U $ml^{-1}$). The cells were trypsinized and duplicate flasks counted at the indicated times. The results of three independent experiments were averaged.

**Figure 2** Inhibition of Colo cell growth by increasing concentrations of IFN. Colo 205 cells were seeded into T25 flasks as described and cultured in RPMI 1640 media and 10% FBS in the presence or absence of the indicated concentrations of IFN and the growth of duplicate flasks was assessed 7 days later. Values are expressed as percent of control cultures that received no IFN. Results are the averages of two independent experiments.

**Figure 3** Effect of IFN on ITS-stimulated growth of Colo 205 cells. Quiescent Colo 205 cells were serum starved for 48 h in the presence or absence of the indicated concentrations of IFN. The cells were then stimulated to grow by addition of ITS (hatched bars) at the concentrations described. Control cultures (solid bars) did not receive ITS. $^{3}$H thymidine incorporation into DNA of quadruplicate wells was measured 16 h later. Results are averages of six independent experiments.

of 100 to 5,000 U ml$^{-1}$ blocked much of the stimulation of DNA synthesis induced by ITS. Incubation of unstimulated cells with IFN also decreased thymidine incorporation. However, this decrease was small (10%) in comparison to the decrease induced in the presence of ITS. Similar results were obtained in initial experiments when cells were assayed 48 h after ITS stimulation suggesting increases in thymidine incorporation were inhibited, rather than delayed, by IFN (data not shown). Decreases in thymidine incorporation were not due to decreases in cell number per well as equal numbers of cells were present at the time of ITS stimulation ($1.6 \pm 0.3 \times 10^5$ for controls *vs.* $1.4 \pm 0.5 \times 10^5$ for IFN treated cultures).

*Flow cytometric analysis*

To ascertain if results obtained by thymidine incorporation methods accurately reflected DNA synthesis, we also estimated the movement of cells through the cell cycle by flow cytometry. The incorporation of thymidine into acid insoluble material has been widely used as a convenient method of assessing the growth inhibitory effect of IFN. However, this technique not only assesses effects on DNA synthesis, but also may reflect alterations in thymidine transport across the plasma membrane, phosphorylation of nucleosides by thymidine kinase, and changes in intracellular pools (Taylor-Papadimitriou *et al.*, 1985*b*). Therefore, cells were grown to confluence and serum starved for 48 h in the presence or absence of IFN (1,000 U ml$^{-1}$). ITS was added and cells harvested 16 h later. Cells were stained with propidium iodide and the numbers of cells in $G_1$, S, or $G_2$/M were assessed as described. Table I shows IFN significantly reduced the number of cells in S phase 16 h after addition of ITS. Flow cytometry data confirmed the inhibition of cell growth observed with thymidine incorporation assays.

*Effect of αIFN on the level of c-*myc *mRNA*

Since IFN$\alpha_2$ inhibited the stimulation of DNA synthesis induced by ITS, we tested whether IFN also impaired the increase of c-*myc* mRNA usually associated with cell replication. The data in Figure 4 reveal this was not the case. As expected, quiescent control cells expressed low levels of c-*myc* transcripts. Increased levels of c-*myc* mRNA were observed in cells treated with ITS only. This increased level of steady state c-*myc* mRNA was also observed in cells treated with both ITS and IFN, under conditions which inhibit cell replication. An increase in steady state c-*myc* RNA levels was also observed in IFN treated cells. Area integration of the densitometric scans of the autoradiogram revealed approximately equal levels of c-*myc* mRNA were found in ITS stimulated cells and cells receiving ITS and IFN (Table II).

**Figure 4** Effect of IFN on the level of c-*myc* mRNA induced by ITS. Confluent Colo 205 cells were placed in RPMI 1640 containing 0.5% FBS and 1,000 U ml$^{-1}$ of IFN if so indicated for 48 h and then stimulated to grow by addition of ITS as described. Total RNA was extracted 2 h later, dot blotted in triplicate onto nitrocellulose at the indicated concentrations, and hybridized with a $^{32}$P-labelled cDNA probe to c-*myc*. Rows 1 = control, 2 = ITS only, 3 = IFN only, 4 = ITS + IFN.

**Table II** Changes in c-*myc* mRNA expression after treatment with ITS and IFN

| *Assay condition* | *Relative level of c-*myc *expression* |
|---|---|
| Control | 1 |
| ITS | 3.3 |
| IFN | 2.2 |
| IFN + ITS | 3.15 |

Confluent Colo 205 cells were exposed to medium containing 0.5% serum (control). Interferon (IFN) (1,000 U ml$^{-1}$) were added to half the flasks for 48 h. Half the flasks in each of the two groups were then exposed to insulin, transferrin and selenium as described (ITS or ITS + IFN). Total RNA was isolated 2 h later. RNA (2 μg) was dot blotted onto nitrocellulose and hybridized to a $^{32}$P labelled c-*myc* probe. The resulting autoradiogram was quantitated by densitometry. Area integrals of these profiles were calculated relative to the level in control cells.

*Effect of time of addition of IFN to quiescent Colo 205 cells*

To determine optimal timing of the IFN treatment, cells were exposed to IFN either before or after addition of ITS at the times indicated (Figure 5). The growth inhibition observed was compared to that obtained by treating cells with IFN for 48 h prior to addition of ITS. IFN$\alpha_2$, added simultaneously with ITS (Time 0), inhibited growth only 50% as well as IFN added for the entire 48 h pretreatment period. IFN added 6 h after addition of ITS failed to inhibit thymidine incorporation. We also varied the length of time of IFN pretreatment. Thymidine incorporation was not as effectively inhibited when cells were pretreated with IFN for only 6 h (Figure 5).

*Effect of increasing concentrations of ITS on IFN mediated growth inhibition*

To determine if increasing concentrations of ITS could overcome the IFN mediated inhibition of cell growth, quiescent Colo cells were stimulated to proliferate by adding increasing concentrations of ITS in the presence or absence of 1,000 units of IFN. The results in Figure 6 indicate that the degree of inhibition of thymidine incorporation was inversely related to the concentration of mitogens. The inhibitory effect of IFN could not be overcome by increasing the concentration of the mitogenic stimulus. Concentrations of ITS 10 times the maximal stimulatory concentration failed to overcome the IFN-induced inhibition of thymidine incorporation.

**Figure 5** Effect of time of addition of IFN on inhibition of DNA synthesis. Colo 205 cells were allowed to grow to confluence and IFN added at the indicated times either prior to exposure to ITS or at the times indicated after exposure to ITS. Thymidine incorporation was assessed 16 h after. Results represent the percent of maximal inhibition observed when cells were exposed to IFN for 48 h prior to addition of ITS. The maximal inhibition observed was $63 \pm 5\%$. Values are the average of three independent experiments (4 wells/point).

**Figure 6** Effect of increasing concentrations of ITS on IFN induced inhibition of growth. Colo 205 cells were seeded at a density of $1 \times 10^5$ cells/well and cultured as described in 24 well plates to achieve quiescence. Cells were then incubated in serum-free media for 48 h in the presence or absence of IFN ($1,000\,IU\,ml^{-1}$). Cells were stimulated with increasing concentrations of ITS and $^3H$ thymidine incorporation in quadruplicate wells determined 16 h later. Values are the averages of three independent experiments.

### Discussion

We have found that pretreatment of quiescent human colon cancer cells with IFN abolishes the mitogenic effect of insulin and transferrin. Our results are in accord with earlier reports indicating the administration of IFN concomitant with serum (Lin *et al.*, 1986), EGF and insulin (Lin *et al.*, 1980; Taylor-Papadimitriou *et al.*, 1981), or PDGF (Tominaga & Lengyel, 1984) blocks $G_0/G_1$-S passage of human and murine fibroblasts. Similarly, growth factor stimulated proliferation of smooth muscle cells (Heyns *et al.*, 1985), or leukaemic cells (Paganelli *et al.*, 1986), is also inhibited by concomitant administration of IFN.

Although pretreatment with IFN diminished the mitogenic effect of ITS, the mechanism of this effect is unknown. The requirement for a long exposure to IFN for effective growth inhibition in our study suggests receptor interactions leading to the antagonistic effect. Similarly, Pfeffer *et al.* (1987) recently demonstrated IFN inhibited insulin-induced growth of Daudi cells. IFN pretreatment of cells reduced binding of insulin to low affinity receptors. Insulin binding was most effectively decreased by a 48 h pre-exposure to IFN. Zoon *et al.* (1986) demonstrated IFN-α inhibited the EGF-stimulated growth of MDBK cells. IFN-α reduced binding of EGF to these cells by decreasing both receptor number and affinity. IFN may have similarly inhibited cell proliferation in our study by decreasing insulin binding.

Alternatively, a secondary interaction between IFN, its receptor, and cytoskeletal elements might have occurred. Cytoskeletal elements, particularly the microtubules, are thought to play a role in signal transduction. IFN can induce tubulin mRNA and interferon's antiviral action can be inhibited by tubulin disrupting agents (Jasny *et al.*, 1985). It is therefore possible that continuous occupancy of the IFN receptor by exogenous ligand resulted in stabilization of the tubulin network, ultimately inhibiting DNA synthesis induced by growth factors. Taylor-Papadimitriou *et al.* (1985*a*) have found that tubulin disrupting agents such as colchicine or nocodazole are very effective at reversing the inhibitory effect of IFN on DNA synthesis.

As expected, exposure of quiescent cells to ITS increased c-*myc* mRNA levels. Although IFN prevented ITS-stimulated thymidine incorporation into DNA, IFN failed to decrease c-*myc* RNA levels down to those observed in unstimulated cells. Tominaga and Lengyel (1984) similarly reported IFN pretreatment of 3T3 cells for 48 hours did not inhibit the ability of PDGF to increase levels of c-*myc* mRNA. Einat *et al.* (1985) found that IFN inhibited growth of HL-60 cells, but failed to reduce steady state levels of c-*myc* mRNA. In contrast, IFN-α produced a decrease of c-*myc* mRNA levels and caused $G_1/G_0$ arrest of Daudi lymphoma cells. In addition we observed increased levels of c-*myc* mRNA in cells treated with IFN alone. Tominaga and Lengyel (1984) found IFN pretreatment of 3T3 cells subsequently exposed to PDGF, resulted in higher levels of c-*myc* mRNA than in cells treated with PDGF only. In contrast to our study, IFN alone did not increase levels of c-*myc* mRNA. The mechanism of this IFN-mediated increase in c-*myc* mRNA levels is not known. Tominaga and Lengyel (1984) suggested that the increase may reflect an IFN-mediated inhibition of labile repressor proteins that regulate steady state levels of c-*myc* mRNA. It is known that IFN differentially regulates protein synthesis. Thus, the increase in levels of c-*myc* mRNA in cells treated with IFN may be a consequence of an impairment of synthesis of these repressor proteins. Thus, IFN blocked thymidine incorporation induced by ITS, but did not prevent an increase in c-*myc* mRNA levels. These findings suggest expression of the c-*myc* oncogene may be a primary consequence of growth factor receptor interaction, rather than a cause or consequence of cell proliferation.

Our results indicated addition of IFN 6 h after administration of insulin failed to inhibit cell growth. Lin *et al.* (1986) similarly demonstrated that addition of IFN 6 h after serum stimulation of 3T3 fibroblasts failed to inhibit cell growth. In contrast, Lin *et al.* (1980) earlier found that IFN rapidly blocks increases in thymidine incorporation even after entry of human fibroblasts into S phase.

The inhibitory effect of IFN on Colo cultures could not be overcome by high concentrations of ITS. The addition of supramaximal concentrations of ITS (10 times the dose required to elicit maximal proliferation) failed to prevent the IFN-induced inhibition of cell growth. The inhibitory effect of IFN is probably not due to a direct competition with ITS. IFN may affect related but not identical cellular pathways as ITS.

In conclusion, we have demonstrated IFN inhibits growth of a colon cancer cell line, in part, by interfering with the ability of ITS to induce growth. Study of the IFN induced regulation of cellular response to insulin and transferrin will provide further insight into the mechanisms of IFN inhibition of cell growth.

### References

BROUTY-BOYE, D., MOGENSEN, K.A. & GRESSER, I. (1985). Effect of long-term treatment of human carcinoma cells with interferon alpha. *Eur. J. Cancer Clin. Oncol.*, **21**, 502.

CHIRGWIN, V.J.M., PRZYBYLA, A.E., MACDONALD, R.F. & KUSTER, W.J. (1979). Isolation of biological active ribonucleic acid from sources enriched in ribonucleases. *Biochem.*, **118**, 5294.

CLEMENS, M.J. & MCMURLAN, M.A. (1985). Regulation of cell proliferation and differentiation by interferons. *Biochem. J.*, **226**, 345.

DELLA-FAVERA, R., MARTIIROTTI, S., GALLO, R.E., ERICKSON, J. & CROCE, C.M. (1985). Translocation and rearrangements of the c-*myc* oncogene located in human B-cell lymphoma. *Science*, **219**, 963.

DENZ, H., LECHLEITNER, M., MARTH, C., DAXENBICHTER, G., GASTL, G. & BRAUNSTEINER, H. (1985). Effect of human recombinant alpha-2- and gamma interferon on the growth of human cell lines from solid tumors and hematologic malignancies. *J. Interferon Res.*, **5**, 147.

EINAT, M., RESNITZKY, D. & KIMCHI, A. (1985). Close link between reduction of c-*myc* expression by interferon and $G_0/G_1$ arrest. *Nature*, **313**, 597.

HEYNS, A., ELDOR, A., VLODAVSKY, I., KAISER, N., FRIDMAN, R. & PANET, A. (1985). The antiproliferative effect of interferon and the mitogenic activity of growth factors are independent cell cycle events. *Exp. Cell Res.*, **161**, 297.

JASNY, B., FRIED, J. & TAMM, I. (1985). The effect of treatment with human interferon B on $^3$H-thymidine uptake and DNA synthesis by colchicine in human fibroblasts. *J. Interferon Res.*, **5**, 239.

KOHASE, M., HENRI, KSEN., DESTEFANO, D., MAY, L.T., VILCEK, J. & SEHGAL, G.B. (1986). Induction of interferon-beta 2 by tumor necrosis factor. A homeostatic mechanism in the control of cell proliferation. *Cell*, **45**, 659.

KRISHAN, A. (1975). Rapid flow cytofluorometric analysis of mammalian cell cycle by propidium iodide staining. *J. Cell Biol.*, **66**, 188.

LIN, S.Z., TS'O, P. & HOLLENBERG, M.D. (1980). The effect of interferon on epidermal growth factor action. *Biochim. Biophys. Res. Commun.*, **96**, 168.

LIN, S.Z., KIKUCHI, T., PLEDGER, W.J. & TAMM, I. (1986). Interferon inhibits the establishment of competence in $G_0$/S phase transition. *Science*, **233**, 356.

OLEZAK, E. & INGLOT, A. (1980). PDGF inhibits antiviral and anticellular action of interferon in synchronized mouse or human cells. *J. Interferon Res.*, **1**, 37.

PAGANELLI, K.A., EVANS, S.S., HAN, T. & OZER, H. (1986). B-cell growth factor-induced proliferation of hairy cell lymphocytes and inhibition by type I interferon *in vitro*. *Blood*, **67**, 937.

PFEFFER, L.M., DONNER, D.B. & TAMM, I. (1987). Interferon-α down-regulates insulin receptors in lymphoblastoid (Daudi) cells. *J. Biol. Chem.*, **262**, 3665.

RESNITSKY, D., YARDEN, A., ZIPORI, D. & KIMCHI, A. (1986). Autocrine β related interferon controls c-*myc* suppression and growth arrest during hematopoietic cell differentiation. *Cell*, **46**, 31.

SCHEITAUER, W., TEMSH, E., SCHIEDER, K., FUNOVICS, A., SCHIESSEL, R. & GRABNER, G. (1985). *In vitro* phase II trial of recombinant interferon alpha$^{-2}$ in gastro intestinal cancer. *Intl J. Cell Cloning*, **3**, 188.

SHIPLEY, G., CHILDS, C., VOEKENOUT, M. & MOSES, H.L. (1984). Differential effects of epidermal growth factor, transforming growth factor and insulin on DNA and protein synthesis and morphology in serum free cultures of AKR-2 B-cells. *Cancer Res.*, **44**, 710.

TAYLOR-PAPADIMITRIOU, J., SHEARER, M. & ROZENGURT, E. (1981). Inhibitory effect of interferon on cellular DNA synthesis: Modulation by pure mitogenic factors. *J. Interferon Res.*, **1**, 401.

TAYLOR-PAPADIMITRIOU. J., BALKWILL, F., ELSWORTH, N. & ROZENGURT, E. (1985*a*). Antiviral and antiproliferative effects of interferon are dissociable. *Virology*, **147**, 405.

TAYLOR-PAPADIMITRIOU, J., ELSWORTH, N. & ROZENGURT, E. (1985*b*). Possible mechanisms of interferon-induced growth inhibition. In *Mediators of Cell Growth and Differentiation*, Ford, R.J. & Maizel, A.L. (eds) p. 283. Raven Press: NY.

TOMINAGA, S. & LENGYEL, P.B. (1984). Interferon alters the pattern of proteins secreted from quiescent and platelet-derived growth factor treated BALB/c 3T3 cells. *J. Biol. Chem.*, **260**, 1975.

ZOON, K.C., KARASAKI, Y., ZUNEDDEN, K., HU, R. & ARNHEITER, H. (1986). Modulation of epidermal growth factor receptors by human α interferon. *Proc. Natl Acad. Sci.*, **83**, 8226.

ZULLO, J., COCHRAN, B., HUANG, A. & STILES, C. (1986). Platelet derived growth factor and double-stranded ribonucleic acids stimulate expression of the same genes in 3T3 cells. *Cell*, **43**, 793.